# Coal Worker's Pneumoconiosis Mimicking Lung Cancer in a 75‐Year‐Old Woman With a History of Breast Cancer: A Clinical Case Report

**DOI:** 10.1002/ccr3.70049

**Published:** 2025-01-02

**Authors:** Yeganeh Pakbaz, Farzan Moodi

**Affiliations:** ^1^ School of Medicine Iran University of Medical Sciences Tehran Iran

**Keywords:** Anthracosis, case report, coal worker pneumoconiosis, lung cancer, progressive massive fibrosis

## Abstract

Pneumoconiosis, caused by inhaling mineral dust, remains a significant occupational disease, despite a declining incidence. Coal workers' pneumoconiosis (CWP), a common subtype, varies in presentation from simple to complicated forms. Differential diagnosis is crucial, especially when CWP manifests as lung masses mimicking malignancy. We present a case of CWP in a 75‐year‐old female with a history of breast cancer, initially suspected of lung cancer due to an incidental mass on chest radiography. Clinical examination, laboratory tests, chest tomography, and biopsy were conducted. The patient, with a history of biomass fuel exposure, presented with a left upper lung mass, initially thought to be lung cancer. Biopsy‐induced hemopneumothorax led to intensive care admission. Histopathology confirmed CWP and conservative management resulted in recovery. Imaging revealed a solid mass in the left upper lung with lymphadenopathy. Histopathology showed carbon‐laden macrophages and anthracosis, consistent with CWP. Imaging modalities, including MRI and FDG‐PET/CT, aid in differentiating CWP from cancer. CWP, mimicking lung cancer, underscores the importance of accurate diagnosis. Imaging features, including nodules with calcifications, guide diagnosis. MRI and FDG‐PET/CT offer valuable insights, albeit with limitations, emphasizing the need for judicious use based on clinical suspicion.

**Summary**

- Coal workers' pneumoconiosis (CWP) poses diagnostic challenges, often mimicking lung cancer.
- Occupational exposure to coal dust increases the risk.
- Accurate diagnosis is crucial.
- Imaging, including MRI and FDG‐PET/CT, aids in differentiation.
- Multidisciplinary collaboration is vital for optimal management, emphasizing awareness among healthcare providers.

## 1 Introduction

Pneumoconiosis is a parenchymal lung disease caused by inhaling various mineral dust, causing parenchymal lung reactions, resulting in fibrotic or nonfibrotic parenchymal lesions [1]. Despite a decline in its incidence rate since 1990; global pneumoconiosis cases have increased globally, from 36,186 in 1990 to 60,555 in 2017. The irreversibility of lung damage and its debilitating nature have made it one of the most important occupational diseases [2]. In China alone, pneumoconiosis accounted for 90% of occupational diseases in 2018 [3]. Common types of pneumoconiosis include asbestosis, silicosis, and coal workers' pneumoconiosis (CWP) [2].

CWP develops due to prolonged exposure to coal dust, leading to varied clinical presentations. On one side, patients have near‐normal lung function with few symptoms and no change in mortality rate, diagnosed as simple CWP, also known as anthracosis. On the other side, patients experience reduced lung function, symptoms such as dyspnea and chronic cough, and a higher risk of mortality rate, diagnosed as complicated CWP, also known as progressive massive fibrosis (PMF) [4]. CWP can manifest as mediastinal lymphadenopathy, fibrosis, nodules, consolidation, or masses requiring differentiation from other malignancies or treatable conditions like tuberculosis [5].

Here, we report a case of CWP in a 75‐year‐old female with a history of cured breast cancer posing a diagnostic challenge due to its presentation as a left upper lobe lung mass resembling lung cancer.

## 2 Case History

**FIGURE 1:**
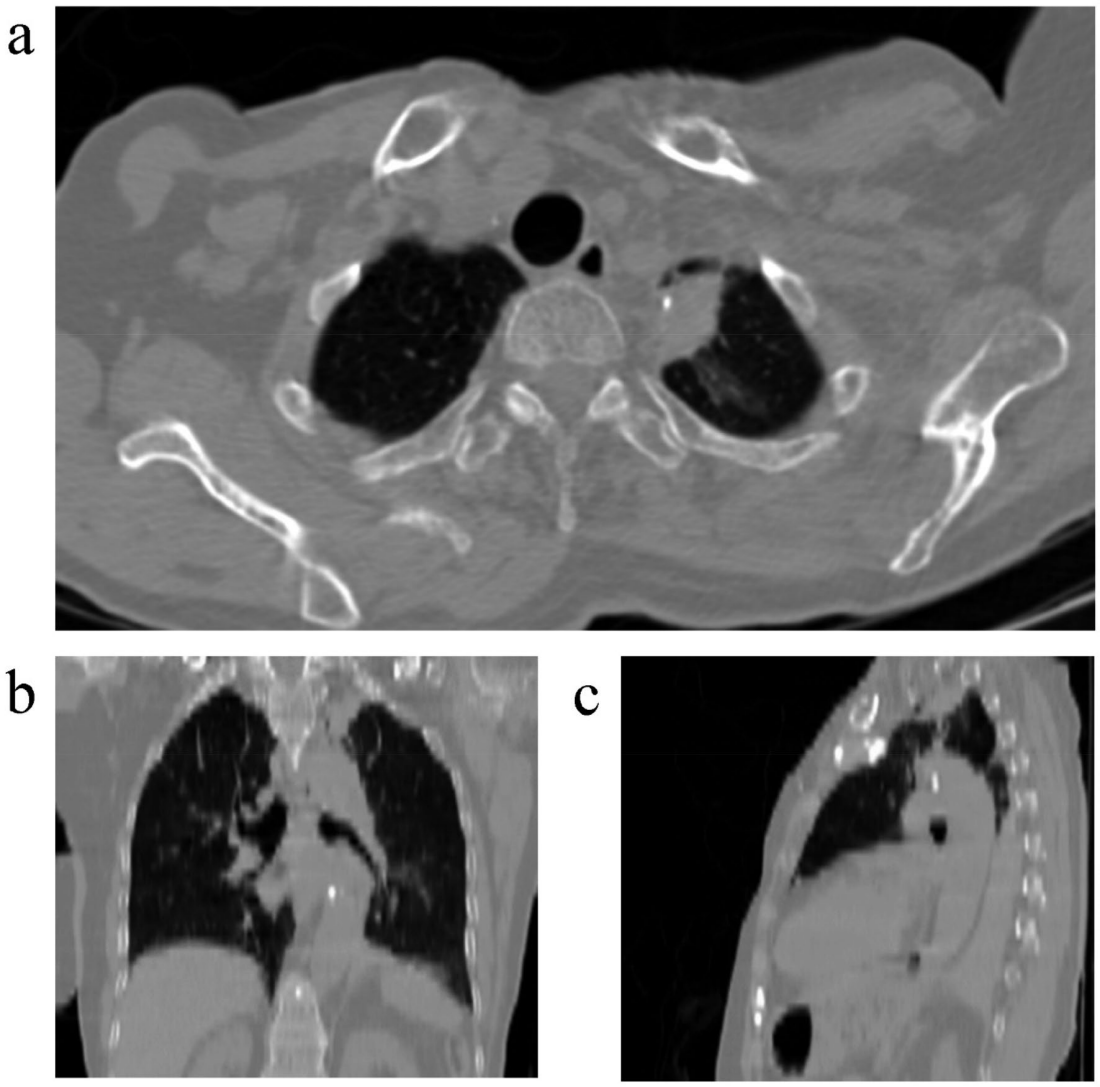
(a) Axial, (b) Coronal, and (c) Sagittal views of CT scan of the pneumoconiosis lesion in the superior left lung lobe, showing a mildly spiculated border with a small area of peripheral calcification.

In December 2020, a 75‐year‐old female with a history of cured breast cancer, hypertension, and more than 30 years of cooking with biomass fuels in poorly ventilated kitchens was referred to the radiology department for further evaluation of an incidental mass found on the chest X‐rays. She reported left shoulder pain persisting for a year and previously was diagnosed and treated as a frozen shoulder. About 11 months after the start of treatment, she did not notice a significant improvement in her symptoms; hence, her family physician ordered a chest x‐ray based on the suspension of possible underlying cancer. She had undergone treatment for left breast cancer 25 years ago, including total mastectomy, adjuvant chemotherapy, and radiotherapy. The chest X‐rays revealed a consolidation favoring lung mass in the left upper hemithorax (Figure 1), and subsequently, she was referred to our hospital. She had no respiratory or constitutional symptoms such as fever, fatigue, weight loss, cough, dyspnea, hemoptysis, etc.

## 3 Methods

Physical examination revealed controlled blood pressure and other vital signs within normal range. Left shoulder movements were painful and restricted. There was a mastectomy scar on the left side of the chest. Auscultation revealed normal lung and heart sounds. No lymphadenopathy was detected. There were no other significant findings. Primary lab tests were as follows: white blood cell 6300/μL, hemoglobulin 12.7 g/dL, platelet count 327,000/μL, urea 40 mg/dL, creatinine 0.9 mg/dL, international normalized ratio (INR) 1 and partial thromboplastin time (PTT) 30 s (reference range: 30–45 s). A chest tomography (CT) scan was ordered. It showed a solid mass with dimensions of 31 x 16 x 22 mm with a spiculated edge containing calcifications in the left upper lobe of the lung.

## 4 Conclusion and Results

A CT‐guided percutaneous core needle biopsy was planned, and the patient was transferred to the operating room. After prepping and draping, multiple nerve blockages in the third and fourth left intercostal spaces with lidocaine 2% were done, and a biopsy specimen was taken, which subsequently caused hemopneumothorax. Immediately, a chest tube was placed, and intensive fluid therapy started. 1.5 L of blood drained into the bottle in minutes. A complete blood count was requested. The patient's hemoglobin decreased from 12.7 to 10.8 g per deciliter, and his platelet count dropped from 327,000 to 242,000/μL. Two units of packed red blood cells were infused, and after stabilization, the patient was transferred to the intensive care unit (ICU).

Histopathology revealed alveolar tissue with a collection of many carbon‐laden macrophages and histiocytes with anthracosis. Fibroblast cells surrounded some areas of necrosis. No atypia or mitosis was detected. Immunohistochemistry (IHC) confirmed CWP diagnosis, with positive CD68 staining and negative panCK staining in these cells.

After one week of conservative management, chest tubes were removed, and the patient was discharged home. The three‐month follow‐up revealed no complications.

## 5 Discussion

Our case highlights the diagnostic challenge posed by CWP, particularly when mimicking lung cancer. Prolonged exposure to biomass fuel in poorly ventilated settings was identified as the underlying cause, highlighting occupational health hazards. There are multiple reports and articles about CWP mimicking lung cancer [6, 7, 8, 9]. It is important to note that CWP can increase the risk of cancer itself. A nationwide study in Taiwan showed that the standardized incidence ratio was 1.12 times more in patients with CWP than in the normal population for all cancers and 1.45 times for mediastinal and lung cancers [10].

Typical Imaging features of CWP include < 5 mm nodules or mass lesions with upper lung zone predominance. Calcifications could be seen in 10%–20% of patients on chest X‐rays and 30% of patients on CT scans. 30% of patients might show mediastinal or hilar lymphadenopathy on CT scans. In complicated form, massive fibrosis presents as large opacities on chest X‐rays. In some cases surrounding emphysematous area could be seen around the lesion on CT scans [1]. Our case demonstrated the typical upper lung zone location with calcifications; however, these findings can also be seen in malignant lesions, along with a spiculated border, which is a feature usually associated with malignancy.

Magnetic resonance imaging (MRI), especially T2‐weighted (T2W) images, seems to be promising in situations where differentiating CWP from cancer is difficult based on CT imaging, as Ogihara and colleagues observed [11]. All lesions diagnosed as PMF consistently exhibit low signal intensity on T2W images (100% sensitivity), whereas 15 out of 16 cancerous lesions had intermediate to high signal intensity on T2W (94% specificity). Nevertheless, differentiating PMF from cancerous lesions based on MRI with contrast enhancement did not appear to be beneficial [11]. Additionally, Zhang et al. looked at another 25 cases with a total of 39 PMF lesions. They discovered that 38 out of 39 lesions had either low or unevenly low signal intensity on T2W and Spectral Presaturation with Inversion Recovery (SPIR). The low signal intensity was compared to the soft muscle in the chest wall. However, only two of the 34 patients with lung cancers or tumors had an uneven equal‐low signal on T2W and SPIR [12].

Another modality for differentiating between pneumoconiosis and cancerous lesions is nuclear imaging [13, 14]. In one study, the use of combined F‐fluorodeoxyglucose (18F‐FDG) positron emission tomography (PET) and CT helped diagnose lung cancer with a sensitivity of 92.8% and a specificity of 87.8%. This combination was better than using FDG or CT alone in terms of reducing their high false positive rates [13].

Both MRI and FDG‐PET/CT have shown great value in differentiating pneumoconiosis from cancer. However, it is important to acknowledge that each imaging modality can produce false positive or negative results, necessitating careful interpretation. Therefore, the use of these modalities might not completely eliminate the need for a biopsy in all cases.

As a wrap‐up for this patient's shoulder pain, it is important to consider that Pancoast tumors can cause shoulder pain due to their invasion of surrounding nerves. In contrast, pneumoconiosis lesions are typically benign. Therefore, we conclude that any shoulder pain experienced by the patient is likely due to the inadequate treatment of the frozen shoulder rather than an underlying malignancy.

In conclusion, this case highlights the diagnostic challenges in distinguishing CWP from lung cancer, particularly when presenting as a suspicious upper lobe lung mass. In our patient, the presence of a spiculated mass with calcifications necessitated thorough investigation to rule out malignancy, ultimately leading to a CWP diagnosis confirmed by histopathology and immunohistochemistry. This underscores the importance of a comprehensive diagnostic approach, particularly in patients with overlapping risk factors, such as a history of biomass fuel exposure and prior cancer. Multidisciplinary collaboration among clinicians, radiologists, and pathologists proved essential in reaching an accurate diagnosis, underscoring that such integration is crucial for optimal patient outcomes. Enhanced awareness of CWP's atypical presentations and potential imaging mimics, including malignancy, can support early and accurate diagnosis, thereby avoiding unnecessary interventions and reducing healthcare burdens.

## Author Contributions

**Yeganeh Pakbaz:** writing – original draft (lead); Investigation (lead); **Farzan Moodi:** conceptualization (lead); writing – original draft (supporting); writing – review and editing (lead).

## Consent

The patient provided written consent to publish this report, following the journal's policy on patient consent.

## Conflicts of Interest

The authors declare no conflicts of interest.

## Data Availability

The data that support the findings of this study are available from the corresponding author upon reasonable request.
